# Effectiveness and safety of high-definition transcranial direct current stimulation in patients with mild cognitive impairment: A randomized, triple-blind, sham-controlled trial

**DOI:** 10.1177/13872877251376547

**Published:** 2025-09-22

**Authors:** Che-Sheng Chu, Hsin-An Chang, Yu-Te Lin, Hsiu-Chu Shen, Chih-Kuang Liang, Ying-Hsin Hsu, Chih-Chuan Pan, Hsin-Ya Kuo, Wei-Zhe Liang, Shiou-Lan Chen, Cheng-Sheng Chen

**Affiliations:** 1 Graduate Institute of Medicine, College of Medicine, Kaohsiung Medical University, Kaohsiung, Taiwan; 2 Department of Psychiatry, Kaohsiung Veterans General Hospital, Kaohsiung, Taiwan; 3 Center for Geriatrics and Gerontology, Kaohsiung Veterans General Hospital, Kaohsiung, Taiwan; 4 Non-invasive Neuromodulation Consortium for Mental Disorders, Society of Psychophysiology, Taipei, Taiwan; 5 Department of Psychiatry, Tri-Service General Hospital, National Defense Medical University, Taipei, Taiwan; 6 Division of Neurology, Department of Internal Medicine, Kaohsiung Veterans General Hospital, Kaohsiung, Taiwan; 7 Department of Business Management, National Sun Yat-Sen University, Kaohsiung, Taiwan; 8 Center for Healthy Longevity and Aging Sciences, National Yang Ming Chiao Tung University, Taipei, Taiwan; 9 Shu-Zen Junior College of Medicine and Management, Kaohsiung, Taiwan; 10 Department of Medical Education and Research, Kaohsiung Veterans General Hospital, Kaohsiung, Taiwan; 11 Department of Pharmacy and Master Program, College of Pharmacy and Health Care, Tajen University, Pingtung, Taiwan; 12 Drug Development and Value Creation Research Center and MSc Program in Tropical, College of Medicine, Kaohsiung Medical University, Kaohsiung, Taiwan; 13 Department of Medical Research, Kaohsiung Medical University Hospital, Kaohsiung, Taiwan; 14 Department of Psychiatry, Kaohsiung Medical University Hospital, Kaohsiung, Taiwan; 15 Department of Psychiatry, College of Medicine, Kaohsiung Medical University, Kaohsiung, Taiwan

**Keywords:** Alzheimer's disease, apolipoprotein E, high-definition transcranial direct current simulation, language, mild cognitive impairment, randomized

## Abstract

**Background:**

High-definition transcranial direct current stimulation (HD-tDCS), a non-invasive brain stimulation technique, has shown potential for improving cognition in patients with mild cognitive impairment (MCI).

**Objective:**

To evaluate whether HD-tDCS enhances cognitive function in individuals with MCI.

**Methods:**

This was a triple-blind, randomized, sham-controlled study. The anodal electrode was placed over the left dorsolateral prefrontal cortex (DLPFC) and surrounded by four cathode electrodes (2 mA for 25 min, daily for 10 sessions). Tests were performed at baseline, after 2-week stimulation, at 1 month, and at 3 months. The primary outcome was global cognition measured by Cognitive Abilities Screening Instrument and secondary outcomes included memory, language, executive function, and attention tests. Group differences were analyzed using linear mixed model (LMM).

**Results:**

Fifty patients with MCI were randomized to the sham (n = 25) and HD-tDCS groups (n = 25). No significant differences were observed between the HD-tDCS and sham groups in global cognition or other neuropsychological measures. However, subgroup analysis revealed a significant three-way interaction between apolipoprotein E (*APOE*) allele 4 status, tDCS condition, and time. *APOE4*-positive patients receiving HD-tDCS showed significantly greater improvement in language function, as measured by the Wechsler Adult Intelligence Scale Fourth Edition vocabulary, compared to *APOE4*-negative patients in the sham group (p = 0.028). Adverse effects were mild and comparable between groups.

**Conclusions:**

HD-tDCS did not enhance global cognition in MCI patients overall. Preliminary findings demonstrated a potential language benefit was observed in *APOE4*-postitive individuals. These exploratory findings warrant further investigation in larger, biomarker-stratified studies.

**Trial registration**

The study was registered at ClinicalTrial.gov under NCT04121156.

## Introduction

Mild cognitive impairment (MCI), a mild neurocognitive disorder, is a borderline state between normal aging and dementia. MCI is a clinically heterogeneous syndrome characterized by subjective cognitive complaints (reported by the individual or an informant), objective evidence of cognitive decline on standardized assessment, preserved functional independence in daily activities, and the absence of a formal diagnosis of dementia.^1^ MCI can be subclassified into amnestic and non-amnestic types, and further into single or multiple domains, depending on the cognitive domains affected. Aging population is a significant factor facilitating the development of MCI. A meta-analysis of 66 studies with 242,804 community-dwelling adults aged 50 years and older showed the overall global prevalence of MCI as 15.6%.^2^ MCI can progress to dementia, primarily in the form of Alzheimer's disease (AD).^3^ The annual progression rate of MCI to dementia is estimated to be 5% to 17%.^3^ Therefore, MCI is viewed as a potential “target” for interventions designed to delay progressions to dementia. Currently, monoclonal antibody treatment is approved by the Food and Drug Administration for patients with MCI and those in early stages of AD. However, the use of monoclonal antibodies is limited owing to cost, availability, and side effects, such as cerebral edema and hemorrhage.^4^ Furthermore, there is limited evidence supporting the efficacy of acetylcholinesterase inhibitors in improving cognition in patients with MCI.^5^ Therefore, there is an urgent need to develop alternative treatment options for patients with MCI.

Accumulating evidence suggests that non-invasive electrical brain stimulation (NIBS) and transcranial direct current stimulation (tDCS) are commonly used techniques with the potential to improve cognitive function in patients with MCI.^6^ tDCS delivers a weak direct electrical current (1 mA-2 mA) to a specific brain region through scalp electrodes and modulates cerebral cortical function by altering cortical excitability.^7^ The left dorsolateral prefrontal cortex (Lt DLPFC) is usually the target region because of its vital role in maintaining executive function and working memory. Empirical evidence supports this approach. For example, Meléndez et al. reported that home-based tDCS applied over the left DLPFC significantly improved global cognition and memory performance in patients with early-stage AD, with sustained improvements at follow-up.^8^ Additional neuroimaging and neurophysiological studies have shown that DLPFC activation is closely associated with task-related cognitive modulation, supporting its use as a neuromodulatory target in mild cognitive impairment and related conditions.^9^ tDCS has the advantages of being relatively safe, simple, and inexpensive to administer compared to other NIBS techniques, such as repetitive transcranial magnetic stimulation.^7^ Clinical research on conventional tDCS, which uses two or more saline-soaked sponge electrodes on the scalp, has shown that anodal tDCS (atDCS) increases the excitability of local neurons and induces subsequent metabolic changes, leading to cognitive improvement in patients clinically diagnosed with MCI based on the Petersen criteria.^1,10^ Several studies have demonstrated that tDCS may improve memory, executive function, and language abilities in this population, both in clinic-based and home-based settings. For instance, one randomized double-blind sham-controlled trial of 58 patients who met the Petersen criteria for MCI showed atDCS (2 mA, 30 min for 10 sessions, twice a week, 5 consecutive weeks) over the Lt DLPFC significantly improved memory recall, verbal fluency, and executive functioning in patients with MCI.^11^ Satorres et al. conducted a randomized, single-blind, placebo-controlled trials and found that home-based atDCS (2 mA, 20 min for 10 consecutive days) over the Lt DLPFC significantly improved cognitive performance in patients with MCI.^12^ Other studies have similarly reported beneficial effects of tDCS on cognitive domains in MCI, including Gomes et al.^11^ Yun et al.^10^ and Sandhya et al.^13^ reinforcing the role of tDCS as a potential alternative intervention. Although conventional tDCS provides potential cognitive benefits in older adults, it may cause relatively non-focal stimulation because of the large size of electrodes (typically 5 × 5 cm or 5 × 7 cm sponges), and the regions between the two electrodes may also be affected.^14^

High-definition (HD)-tDCS is topographically more accurate and can provide increased current density to penetrate deeper cortical regions based on its unique montage design. HD-tDCS, also called 4 × 1 HD-tDCS, employs five-ring silver electrodes (circular shape and radius of 10 mm) with an active electrode placed over the target region surrounded by four return electrodes.^15^ Currently, few studies have used HD-tDCS for the treatment of MCI.^16–19^ Notably, three studies diagnosed MCI,^16,18,19^ and one study specifically focused on the diagnosis of amnestic MCI^17^; all four studies based their diagnoses solely on clinical assessments and neuropsychological testing, without the use of biomarker-based evidence. The studies respectively employed the Petersen criteria,^1^ the National Institute on Aging and Alzheimer's Association,^20^ and the Diagnostic and Statistical Manual of Mental Disorders, Fifth Edition.^21^ A single blind, sham-controlled study of 30 patients with MCI who received HD-tDCS (1 mA, 20 min for 10 sessions, once daily, 2 consecutive weeks) over the Lt DLPFC showed that active HD-tDCS improved global cognitive function, as measured via the Montreal Cognitive Assessment (MoCA) but not the Mini-Mental State Examination (MMSE), compared to those receiving sham stimulation.^18^ Another double blind, cross-over study of 20 patients with MCI received HD-tDCS (2 mA, one session, 20 min) over right parietal cortex showed that HD-tDCS may normalize functional network segregation, but no changes were detected at the cognitive performance.^17^ Finally, a recent double-blind sham-controlled study of 60 patients with MCI who received HD-tDCS (2 mA, 20 min for 10 sessions, once daily, for 2 consecutive weeks) over the Lt DLPFC or dominant anterior temporal lobe showed that active HD-tDCS improved cognitive function, as measured using MoCA compared to sham stimulation.^16^ Notably, Rezakhani et al. conducted a different montage with four anodal electrodes over the brain area and one cathodal electrode placed in the contralateral supraorbital region.^16^ Taken together, it remains challenging to draw firm conclusions regarding the efficacy of HD-tDCS on cognitive function in patients with MCI owing to those prior studies who often had small sample sizes, single-blind designs, non-standard electrode montages, or atypical stimulation protocols. Our study addresses these limitations by employing a triple-blind design, a larger sample size, a standard 4 × 1 HD-tDCS montage over the Lt DLPFC, and more comprehensive cognitive assessments.

This study aimed to investigate the effects of repeated anodal HD-tDCS on the cognitive function of patients with MCI. We focused not only on global cognitive functions but also on several specific cognitive functions, such as memory, executive function, language function, and working memory. We hypothesized that active anodal HD-tDCS would improve cognitive function in patients with MCI compared to those receiving sham stimulation.

## Methods

### Ethical statement

This study was approved by the Ethics Review Boards of Kaohsiung Veterans General Hospital (KSVGH20-CT1-11) and the Taiwan Food and Drug Administration (TFDA 1086614707). All the procedures met the requirements of the Declaration of Helsinki. Participants were included only after obtaining informed consent following a detailed explanation of the study procedures. The study was registered at ClinicalTrial.gov under NCT04121156.

### Subject and setting

This study was conducted at the Kaohsiung Veterans General Hospital, Kaohsiung, Taiwan. The trial was a two-arm, triple-blind (participants, stimulation operators, and outcome assessor/raters), prospective, 14-week study of two groups of patients with MCI recruited from a memory clinic, which was performed in a single medical center from September 2020 to August 2024. Patients with MCI were diagnosed by an expert psychiatrist or neurologist based on Petersen criteria.^1^ The inclusion criteria were as follows: (a) age between 60 and 85 years; (b) complaints of memory decline (confirmed by others or relatives who were familiar with the participant); (c) cognitive impairment that did not interfere with the capacity for independence in everyday activities; (d) clinical dementia rating scale equal to 0.5 or MMSE score greater than 24; € no dementia diagnosis; and (f) right-handed. Additionally, brain imaging, such as brain computed tomography (CT) or magnetic resonance imaging (MRI), was performed to ensure that the patients did not have comorbidities other than MCI. Specifically, we excluded patients with evidence of brain tumors, stroke, normal pressure hydrocephalus, traumatic brain injury, or other major neurodegenerative disorders (e.g., Parkinson's disease, frontotemporal dementia). Furthermore, psychiatric comorbidities (e.g., major depressive disorder, generalized anxiety disorder, schizophrenia) and those receiving psychotropic medications were excluded to minimize confounding cognitive effects. Finally, we excluded patients with MCI (a) having contraindications for tDCS; (b) concurrent treatment with psychotropic agents such as antidepressants, antipsychotics, anxiolytics, and benzodiazepines; (c) with a history of chemotherapy or radiotherapy; (d) Hamilton depression rating scale score 17 items (HAMD-17) greater than 12; and € participants who received fewer than seven treatment sessions out of the planned ten were excluded to ensure intervention adherence.

### Intervention

**Figure 1.**
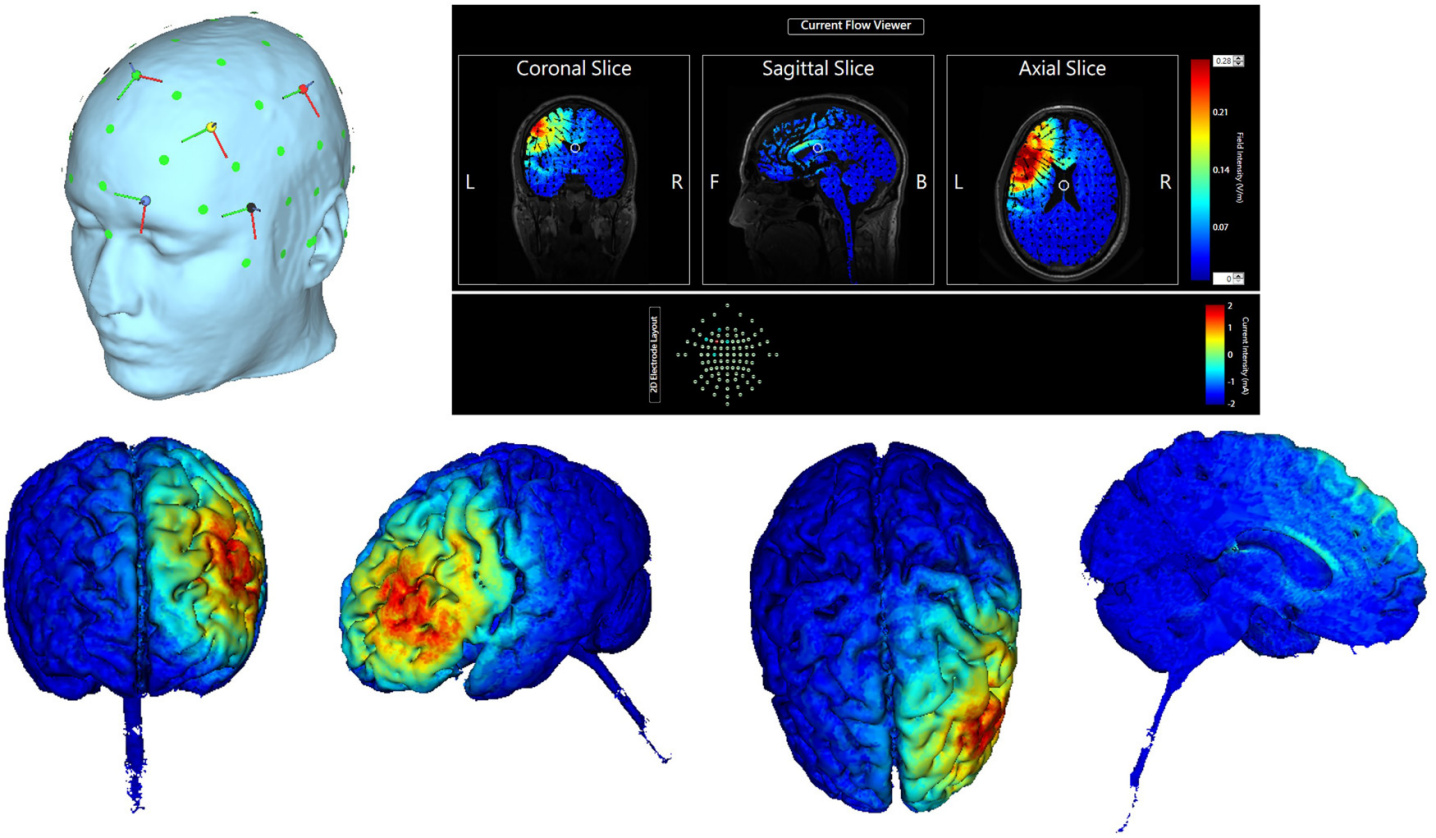
(A) Electrode placement. (B, C1-1C4) Electric field simulation. Numerical computation of electric field.

Fifty patients diagnosed with MCI received a 2-week intervention with either active or sham stimulation. An Eldith DC stimulator (Neuroconn DC Stimulator Plus, GmbH, Ilmenau, Germany) was used for stimulation. According to the 10–20 standard international system for electroencephalogram electrode placement, the anodal electrode was placed over the Lt DLPFC, which corresponded to the F3 location. The anode was surrounded by four cathodes, which were placed at Fp1, Fz, C3, and F7 (Figure 1A). Stimulation was applied at an intensity of 2 mA for 25 min once daily for 2 weeks on 10 consecutive weekdays. In the sham stimulation, a 2 mA current was turned on for 30 s and then ramped down to 0 mA for the remainder of the 25-min duration. A numerical computation of the electric field was performed (Figure 1B and C). All patients were asked whether they had received active or sham HD-tDCS treatment 1 h after the first stimulation session. Throughout the trial, the use of anti-dementia medications (such as acetylcholinesterase inhibitors and N-methyl-D-aspartate receptor antagonists) and psychotropic agents (including antidepressants, antipsychotics, anxiolytics, and benzodiazepines) was prohibited to minimize potential confounding effects on cognitive function and reduce bias in the study outcomes. All such medications were not allowed at least three months prior to the first stimulation session and remained restricted until the completion of the final follow-up at week 14.

### Participants screening and enrollment

**Figure 2.**
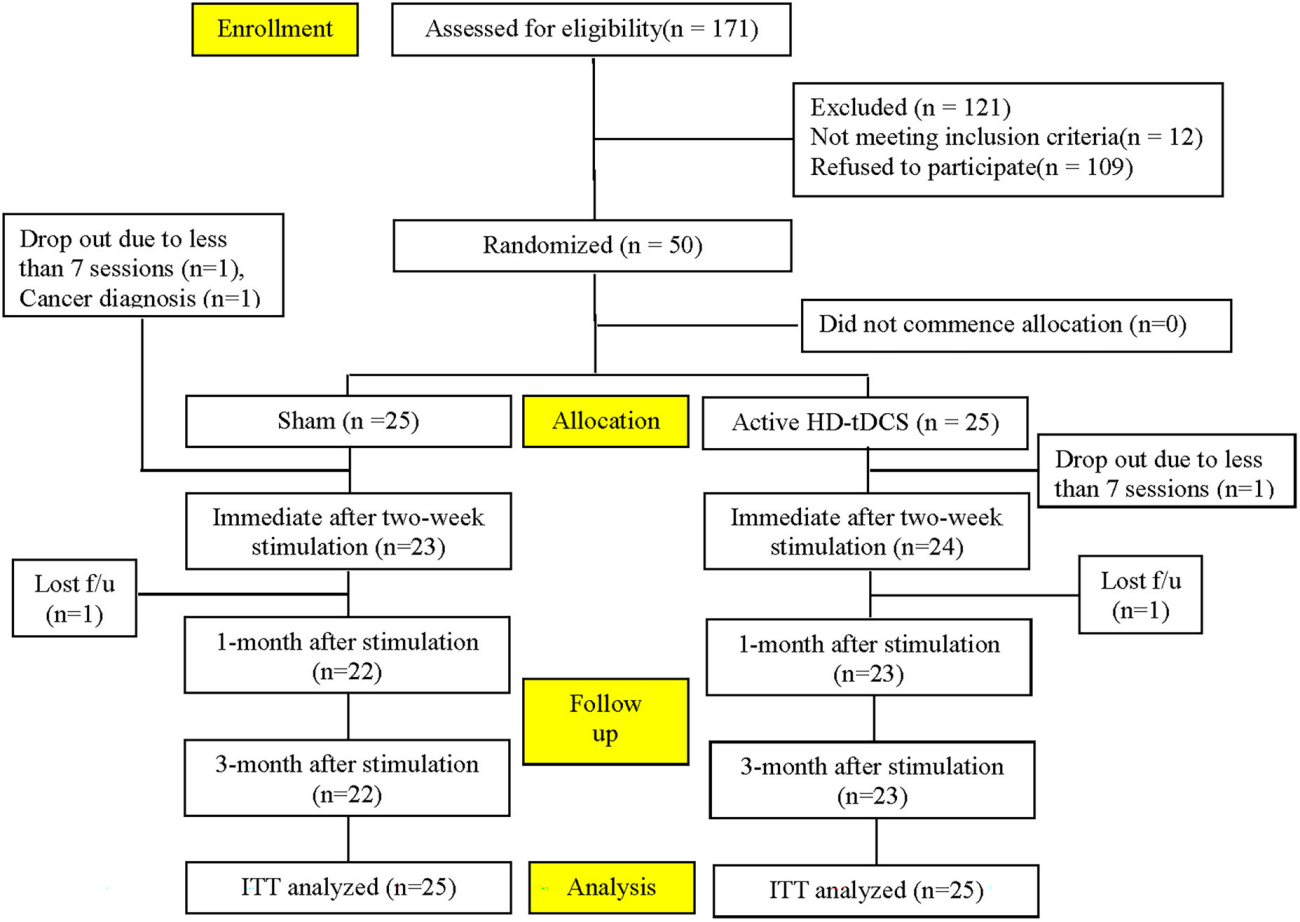
CONSORT flow chart diagram.

Initially, 171 individuals diagnosed with MCI were recruited for the trial. Nevertheless, 121 patients were ineligible based on the inclusion criteria and were excluded from the study. Additionally, two participants withdrew from the study before completing the entire treatment, having received fewer than seven stimulation sessions. One was initially assigned to the sham group and the other to the HD-tDCS treatment group. The final sample comprised 50 patients with MCI, with 25 receiving sham treatment and 25 undergoing HD-tDCS treatment. A CONSORT flowchart diagram is shown in Figure 2.

A substantial proportion of potential participants with MCI declined enrollment due to the considerable distance and economic burden associated with attending daily on-site intervention sessions. Specifically, the time commitment and transportation costs—particularly for those residing in remote or suburban areas—posed significant barriers to participation. For elderly individuals, these demands often translated into undue physical and cognitive strain. In addition, a notable subset of candidates refused participation due to a lack of perceived cognitive deficits,^22^ which is a well-recognized characteristic in MCI populations and often contributes to low treatment-seeking behavior. These observations underscore the practical limitations of hospital-based neuromodulation trials and highlight the importance of developing home-based tDCS protocols.^8,12^ Such approaches may enhance the feasibility and scalability of future studies by improving accessibility, promoting participant adherence, and expanding recruitment to broader, more representative populations beyond tertiary care settings.

### Outcome measures

The primary outcome was the change in global cognitive function during the study period using the Cognitive Abilities Screening Instrument (CASI).^23^ CASI is a comprehensive neuropsychological test containing nine domains: long-term memory, short-term memory, attention, concentration/mental manipulation, orientation, abstraction/judgment, language, visual construction, and list-generating fluency. The scores range from 0 to 100, with lower scores indicating greater severity of cognitive impairment.

For the secondary outcome measurements, we applied several standardized neuropsychological tests to assess various cognitive domains. The Wechsler Memory Scale (WMS-III) verbal paired associates and visual reproduction subtests were used to assess verbal and visual memory, respectively. The Wisconsin Card Sorting Test (WCST) evaluated working memory and cognitive flexibility. The Frontal Assessment Battery (FAB) was used to assess executive function. Attention and processing speed were measured using the digit span and digit symbol coding subtests of the Wechsler Adult Intelligence Scale-Fourth Edition (WAIS-IV), respectively. Language function was evaluated using the vocabulary subtest of the WAIS-IV. Data from the self-reported Beck Depression Inventory, Beck Anxiety Inventory, and Subjective Cognitive Decline Questionnaire were collected during the trial.

Side effects were assessed after each HD-tDCS session using open-ended questions about physical discomfort, followed by close-ended questions addressing common HD-tDCS side effects, such as scalp pain, discomfort at the application sites, including tingling, itching, burning sensations, and skin redness.^24^

All examinations (including objective cognitive tests and self-reported scales) were administered by a trained rater who was blinded to the group assignment at baseline, after 2-week stimulation, at 1 month (week 6), and 3 month (week 14) of follow-up. All participants were reimbursed for their transportation costs and the time spent at each follow-up appointment (totally USD 12.2 for the trial).

### Randomization and blinding

Randomization was performed using a computer-generated randomization list with a 1:1 allocation based on the permuted block method using a random number generator. A research assistant who was independent of the enrollment, stimulation, and evaluation concealed the allocation sequence. Each participant eligible for the trial was assigned a study code prior to the first stimulation. The operator was responsible for entering a code provided by a third party into the stimulation device. The device was pre-programmed to automatically deliver either active or sham stimulation based on the code. Therefore, the operator was not aware of whether the participants received active stimulation or not. To assess the integrity of the blinding procedure, participants were surveyed at the end of the stimulation (week 2) regarding their beliefs about the treatment condition they received (i.e., active versus sham). An independent research statistician completed all statistical analyses after the trial completion.

### Sample calculation

The sample size was estimated using data from a previous component network meta-analysis study on tDCS in the treatment of AD and MCI to compare the baseline and endpoints of the active treatment group. The pooled effect size of atDCS on general cognition was 0.56.^6^ Using these parameters, G*power 3.1.9.4 was employed to calculate the sample size for an ANOVA (repeated measures, within-between interaction) for the primary outcome measure, with an effect size of f = 0.56, alpha error probability = 0.05, power (1- beta error probability) = 0.95, and two groups. The calculated total sample size required is only 10. Initially, the strictest definition was set with an effect size of 0.2, and we obtained a calculated total sample size of 60 (N = 30 in each group) using the same setting as in the G* Power 3.1.9.4. However, owing to the impact of COVID-19 and a shortage of study funds, the trial was terminated early. Finally, we recruited 50 participants (N = 25 in each group) to obtain an effect size of at least 0.22, which was considered a small effect size.

### Statistical analysis

Baseline characteristics were compared between the sham stimulation and HD-tDCS stimulation groups using independent sample t-tests for continuous variables (e.g., age and CASI score) and Fisher's exact tests for categorical variables (e.g., sex and apolipoprotein E [*APOE*] 4 carriers). First, cognitive function changes (e.g., CASI, WMS, and WAIS) from pre-to post-intervention measurements (i.e., 2nd week, 6th week, and 14th week) between the two treatment groups were compared using a linear mixed model (LMM). LMM included the main effects of the treatment groups (sham stimulation group versus HD-tDCS stimulation group), time points (classified as categorical variables), and two-way interaction terms between the treatment groups and time points. The difference between the baseline and a specific post-intervention measurement within each treatment group was tested using the contrast of simple main effects in the LMM. Furthermore, the differences in cognitive function changes between treatment groups across various subgroups (i.e., sexes, *APOE4* carriers, and cognitive status [MMSE ≥24 versus MMSE <24]) were assessed using three-way interaction terms involving treatment groups, time points, and the respective subgroup variables. The random intercept in the LMM was set to account for repeated measurements in each subject. Data was analyzed using SPSS version 26 (IBM SPSS Inc., Chicago, IL, USA). A two-sided significance less than 0.05 was considered statistically significant.

## Results

### Characteristics of the study population, including demographic and clinical factors

The baseline demographic characteristics of the sham and active HD-tDCS groups are shown in Table 1. The initial demographic and clinical profiles showed no statistically significant differences between the sham and active stimulation groups, except for the WMS-III visual paired associates (immediate recall) (p = 0.045), where a significant difference was observed.

**Table 1.** Baseline characteristics of participants in the sham stimulation and active HD-tDCS groups.

Variables	Available *N*	Total	Sham (*n* = 25)	Active (*n* = 25)	*p*
Demographics					
Male sex	50	25 (50.0)	13 (52.0)	12 (48.0)	0.777
Age, y	50	73.7 ± 6.3	74.3 ± 6.6	73.2 ± 6.0	0.547
Education level, y	50	10.7 ± 4.4	10.7 ± 3.9	10.7 ± 4.9	0.975
Genetic polymorphisms					
*APOE4*	47	12 (25.5)	5 (21.7)	7 (29.2)	0.559
BDNF type	29				0.909
GG		8 (27.6)	4 (26.7)	4 (28.6)	
GA + AA		21 (72.4)	11 (73.3)	10 (71.4)	
Cognitive and neuropsychological assessments					
HAMD score	50	2.5 ± 2.7	3.0 ± 3.3	2.0 ± 2.0	0.202
MMSE score	50	24.6 ± 3.6	24.5 ± 4.1	24.7 ± 3.2	0.849
CASI score	50	78.9 ± 10.3	77.6 ± 10.4	80.2 ± 10.2	0.390
WMS-III-Visual paired association-immediate	50	3.6 ± 5.2	2.2 ± 3.3	5.1 ± 6.4	**0**.**045**
WMS-III-Visual paired association-delay	50	0.9 ± 1.5	0.7 ± 1.2	1.2 ± 1.8	0.319
WMS-III-visual reproduction-immediate	50	11.5 ± 6.2	11.7 ± 7.1	11.2 ± 5.4	0.772
WMS-III-visual reproduction-delay	50	9.6 ± 6.9	9.8 ± 6.9	9.3 ± 7.0	0.808
WAIS-IV-digit span	50	19.9 ± 5.4	20.1 ± 5.0	19.8 ± 5.8	0.855
WAIS-IV-digit symbol coding	50	15.0 ± 8.1	14.1 ± 8.2	15.9 ± 8.0	0.447
WAIS-IV-vocabulary	50	17.6 ± 9.9	17.2 ± 10.0	18.0 ± 10.1	0.801
WCST: total trials	50	47.8 ± 14.8	44.8 ± 13.2	50.7 ± 15.9	0.159
FAB	50	11.7 ± 3.0	11.3 ± 3.3	12.1 ± 2.7	0.327
SCD-Q	50	8.2 ± 3.9	8.3 ± 4.0	8.1 ± 3.8	0.858
BDI	50	5.0 ± 7.4	6.3 ± 9.4	3.7 ± 4.4	0.222
BAI	50	4.0 ± 6.1	5.4 ± 7.7	2.6 ± 3.8	0.119

*APOE4*: apolipoprotein E4 allele; GG: homozygous for the G allele; GA: heterozygous for the G allele; AA: homozygous for the A allele; HAMD: Hamilton Depression Rating Scale; MMSE: Mini-Mental State Examination; CASI: Cognitive Abilities Screening Instrument; WMS-III: Wechsler Memory Scale-Third Edition; WMS-IV: Wechsler Memory Scale-Fourth Edition; FAB: Frontal Assessment Battery.

Data are presented as frequency (percentage) or mean ± standard deviation.

Bold type indicates significant difference.

### Clinical outcomes: global cognition

In the sham stimulation group, the average CASI score increased from 77.6 ± 10.4 at baseline to 79.1 ± 10.5 after 2-week stimulation period. In the HD-tDCS stimulation group, the average CASI score increased from 80.2 ± 10.2 at baseline to 81.4 ± 11.4 after 2-week stimulation period. A significant main effect of time on increasing CASI scores was identified in both the sham and HD-tDCS groups after the 6-week and 14-week follow-up periods (p < 0.05), but the increase in CASI scores was independent of the group, i.e., the improvements were not specific to either sham or HD-tDCS stimulation (Table 2). Specifically, this indicated that compared to baseline, global cognitive function in the 6-week and 14-week time periods increased independently of whether participants received sham stimulation or HD-tDCS stimulation. The interaction effect between time and group was not significant at any time point (compared to baseline), indicating that HD-tDCS stimulation did not improve global cognitive function in patients with MCI.

**Table 2.** The results of linear mixed models between the sham stimulation and the active HD-tDCS groups.

Variable	Sham (*n* = 25)	HD-tDCS (*n* = 25)	Group difference of the mean change from baseline (95% CI)	*p* for interaction	Between time		*p* between group
					p* of group A	p* of group B	
Primary outcome: CASI score							
Baseline	77.6 ± 10.4	80.2 ± 10.2	Reference	-	-	-	0.404
2 weeks	79.1 ± 10.5	81.4 ± 11.4	0.20 (−3.22, 3.62)	0.908	0.246	0.318	0.442
6 weeks	80.3 ± 10.5	83.2 ± 11.8	−0.41 (−3.83, 3.01)	0.812	**0**.**031**	**0**.**013**	0.332
14 weeks	81.4 ± 10.2	83.2 ± 10.4	0.74 (−2.68, 4.15)	0.671	**0**.**003**	**0**.**015**	0.554
WMS-III-Visual paired association-immediate							
Baseline	2.2 ± 3.3	5.1 ± 6.4	Reference	-	-	-	0.118
2 weeks	4.2 ± 5.0	7.2 ± 8.1	−0.12 (−2.17, 1.93)	0.908	**0**.**007**	**0**.**005**	0.104
6 weeks	5.0 ± 4.8	8.1 ± 8.4	−0.16 (−2.21, 1.89)	0.878	**<0**.**001**	**<0**.**001**	0.099
14 weeks	5.8 ± 5.9	9.2 ± 9.1	−0.48 (−2.53, 1.57)	0.645	**<0**.**001**	**<0**.**001**	0.069
WMS-III-Visual paired association-delay							
Baseline	0.7 ± 1.2	1.2 ± 1.8	Reference	-	-	-	0.456
2 weeks	1.2 ± 1.7	1.7 ± 2.3	−0.04 (−0.83, 0.75)	0.921	0.093	0.069	0.417
6 weeks	1.6 ± 1.6	2.4 ± 3.1	−0.32 (−1.11, 0.47)	0.427	**0**.**002**	**<0**.**001**	0.199
14 weeks	1.5 ± 1.9	2.3 ± 2.3	−0.36 (−1.15, 0.43)	0.371	**0**.**005**	**<0**.**001**	0.177
WMS-III-visual reproduction-immediate	1						
Baseline	11.7 ± 7.1	11.2 ± 5.4	Reference	-	-	**-**	0.827
2 weeks	13.7 ± 10.9	14.4 ± 7.0	−1.16 (−4.68, 2.36)	0.516	0.115	**0**.**013**	0.788
6 weeks	15.3 ± 9.5	14.8 ± 7.4	0.00 (−3.52, 3.52)	1.000	**0**.**005**	**0**.**005**	0.827
14 weeks	16.1 ± 10.3	15.1 ± 8.0	0.48 (−3.04, 4.00)	0.788	**<0**.**001**	**0**.**002**	0.674
WMS-III-visual reproduction-delay							
Baseline	9.8 ± 6.9	9.3 ± 7.0	Reference	-	-	-	0.855
2 weeks	12.6 ± 10.5	12.2 ± 9.0	−0.04 (−4.15, 4.07)	0.985	0.055	0.052	0.867
6 weeks	12.7 ± 10.1	13.8 ± 8.9	−1.64 (−5.75, 2.47)	0.432	0.052	**0**.**003**	0.658
14 weeks	14.2 ± 12.4	13.6 ± 7.9	0.04 (−4.07, 4.15)	0.985	**0**.**004**	**0**.**004**	0.843
WAIS-IV-digit span							
Baseline	20.1 ± 5.0	19.8 ± 5.8	Reference	-	-	-	0.869
2 weeks	19.7 ± 5.1	20.8 ± 6.6	−1.44 (−3.19, 0.31)	0.107	0.524	0.099	0.495
6 weeks	20.0 ± 5.9	21.2 ± 6.6	−1.56 (−3.31, 0.19)	0.081	0.848	**0**.**023**	0.451
14 weeks	21.5 ± 5.9	21.4 ± 6.7	−0.16 (−1.91, 1.59)	0.857	**0**.**023**	**0**.**012**	0.944
WAIS-IV-digit symbol coding							
Baseline	14.1 ± 8.2	15.9 ± 8.0	Reference	-	-	-	0.494
2 weeks	15.4 ± 9.9	18.0 ± 8.8	−0.76 (−3.46, 1.94)	0.579	0.174	**0**.**033**	0.328
6 weeks	15.8 ± 10.3	18.8 ± 9.1	−1.28 (−3.98, 1.42)	0.351	0.092	**0**.**003**	0.238
14 weeks	16.4 ± 9.0	19.6 ± 9.0	−1.44 (−4.14, 1.26)	0.294	**0**.**022**	**<0**.**001**	0.215
WAIS-IV-vocabulary							
Baseline	17.2 ± 10.0	18.0 ± 10.1	Reference	-	-	-	0.804
2 weeks	16.5 ± 9.2	18.7 ± 10.0	−1.48 (−4.43, 1.47)	0.323	0.472	0.496	0.449
6 weeks	17.4 ± 9.8	19.7 ± 10.7	−1.60 (−4.55, 1.35)	0.285	0.880	0.097	0.425
14 weeks	17.6 ± 10.4	20.4 ± 11.6	−2.04 (−4.99, 0.91)	0.173	0.733	**0**.**024**	0.342
WCST: total trials							
Baseline	44.8 ± 13.2	50.7 ± 15.9	Reference	-	-	-	
2 weeks	49.2 ± 15.4	51.2 ± 17.0	3.88 (−3.38,11.14)	0.293	0.096	0.854	0.667
6 weeks	47.8 ± 19.0	52.4 ± 17.6	1.32 (−5.94, 8.58)	0.720	0.257	0.529	0.333
14 weeks	49.8 ± 19.5	46.9 ± 15.5	8.80 (1.54, 16.06)	**0**.**018**	0.056	0.146	0.544
FAB score							
Baseline	11.3 ± 3.3	12.1 ± 2.7	Reference	-	-	-	0.305
2 weeks	11.4 ± 2.9	12.3 ± 2.7	−0.08 (−1.23, 1.07)	0.891	0.771	0.627	0.262
6 weeks	12.4 ± 2.9	12.8 ± 2.9	0.44 (−0.71, 1.59)	0.450	**0**.**005**	0.082	0.625
14 weeks	12.3 ± 2.8	13.3 ± 2.9	−0.12 (−1.27, 1.03)	0.837	**0**.**012**	**0**.**005**	0.242
SCD-Q							
Baseline	8.3 ± 4.0	8.1 ± 3.8	Reference		**-**	**-**	0.858
2 weeks	8.5 ± 4.2	7.2 ± 3.6	1.04 (−0.57, 2.65)	0.203	0.781	0.128	0.269
6 weeks	8.0 ± 4.2	7.7 ± 3.9	0.16 (−1.45, 1.77)	0.844	0.627	0.445	0.748
14 weeks	7.8 ± 4.2	8.5 ± 3.6	−0.96 (−2.57, 0.65)	0.240	0.332	0.488	0.498
BDI-II							
Baseline	6.3 ± 9.4	3.7 ± 4.4	Reference		**-**	**-**	0.144
2 weeks	4.5 ± 7.1	3.0 ± 4.1	−1.04 (−3.23, 1.15)	0.350	**0**.**026**	0.360	0.385
6 weeks	4.4 ± 6.5	3.4 ± 4.5	−1.56 (−3.75, 0.63)	0.162	**0**.**016**	0.647	0.567
14 weeks	4.8 ± 6.9	3.2 ± 4.4	−1.04 (−3.23, 1.15)	0.350	0.054	0.541	0.385
BAI							
Baseline	5.4 ± 7.7	2.6 ± 3.8	Reference		**-**	**-**	**0**.**033**
2 weeks	3.5 ± 5.4	1.5 ± 3.1	−0.72 (−2.65, 1.21)	0.462	**0**.**007**	0.095	0.117
6 weeks	2.5 ± 3.4	1.7 ± 3.2	−1.96 (−3.89, −0.03)	**0**.**047**	**<0**.**001**	0.185	0.550
14 weeks	3.3 ± 4.1	1.7 ± 3.1	−1.16 (−3.09, 0.77)	0.237	**0**.**003**	0.185	0.220

CASI: Cognitive Abilities Screening Instrument; WMS-III: Wechsler Memory Scale-Third Edition; WMS-IV: Wechsler Memory Scale-Fourth Edition; FAB: Frontal Assessment Battery.

Data were presented as mean ± standard deviation.

Bold type indicates significant difference.

*The contrast of simple main effect within the linear mixed model with two-way interactions.

### Clinical outcomes: subdomain cognition

For the subdomain of cognition, visual memory, as measured using WMS-III-verbal paired associates (immediate and delayed recall), showed a significant increase over time compared to baseline in both groups, but the difference was independent of the sham or HD-tDCS group. Several delayed cognitive improvements were observed at the 6-week or 14-week follow-up periods after completing 10 sessions, particularly in the HD-tDCS group. These improvements were found in the following areas: WMS-III-verbal paired association (delayed recall, verbal memory), WMS-III-visual reproduction (immediate and delayed recall, visual memory), WAIS-IV-digit span (attention), WAIS-IV-digit symbol coding (processing speed), WAIS-IV-vocabulary (language), and FAB (executive function) (all p value < 0.05). However, the interaction effect between time and group was not significant at any time point for the tested subdomain cognition (Table 2). We found that patients with MCI receiving HD-tDCS had significantly fewer total trials of the WCST at week 14 than those receiving sham stimulation (p = 0.018); however, there was no interaction effect between time and group regarding the other domains of the WCST at any time points (Supplemental Table 1).

### Subgroup analysis: global cognition and subdomain cognition

**Figure 3.**
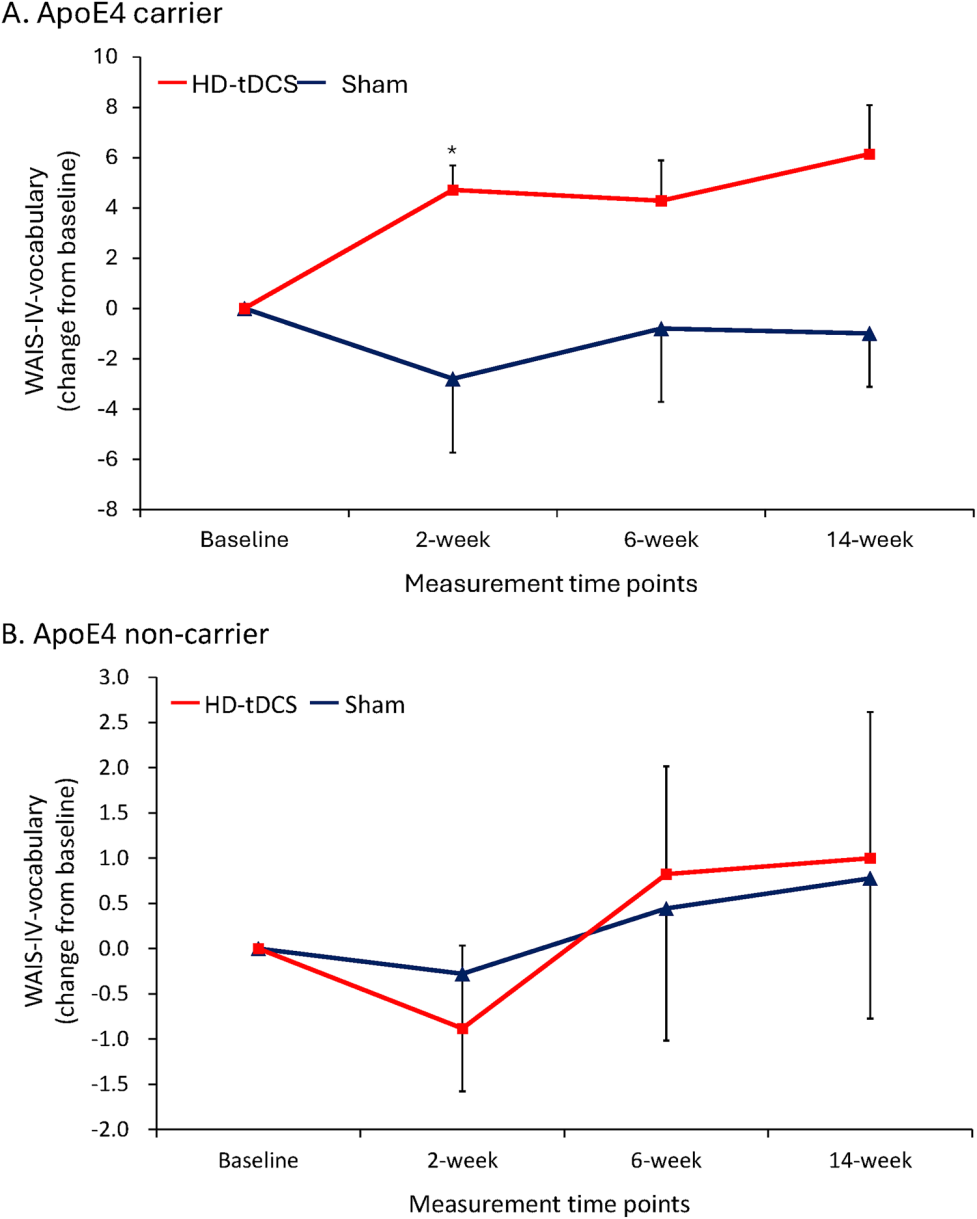
The WAIS-IV-vocabulary scores of participants between the sham stimulation and active HD-tDCS stimulation groups with *APOE4* carrier (A) and without *APOE4* carrier (B). Data are presented as mean and standard error of the mean. “*” indicates the improvement from baseline to the specific time point was significantly different between the treatment groups. The three-way interaction effect was significant at 2 weeks (p = 0.028). WAIS-IV: Wechsler adult intelligence scale; HD-tDCS: high-definition transcranial direct current stimulation; *APOE4*: apolipoprotein E4 allele.

To address the issue of heterogeneity across patients with MCI, we conducted subgroup analysis by dividing into *APOE4*-positive patients and *APOE4*-negative patients, patients with MMSE ≥24 or <24. BDNF genotype status was not included in the subgroup analysis because of the availability of only 29 participants. As shown in Table 3, the LLM revealed a significant three-way interaction between *APOE4* status, tDCS condition, and time of testing, demonstrating an improvement in language function as measured through the WAIS-IV-vocabulary at 2 weeks (p = 0.028). *APOE4*-positive patients who received HD-tDCS for 10 daily sessions showed improved language function, as measured via WAIS-IV vocabulary, than *APOE4*-negative patients receiving sham stimulation (Table 3, Figure 3A and B). None of the three-way interaction effects were significant in the subgroup analysis of global cognitive function or other subcognitive domains (except WAIS-IV vocabulary) (Table 3, Supplemental Table 2).

**Table 3.** Subgroup analysis: the results of linear mixed models in the sham stimulation and active HD-tDCS groups.

	*APOE4*+ (*n* = 12)			*APOE4*- (*n* = 35)			
Variable	Sham (*n* = 5)	HD-tDCS (*n* = 7)	Group difference of the mean change from baseline (95% CI)	Sham (*n* = 18)	HD-tDCS (*n* = 17)	Group difference of the mean change from baseline (95% CI)	*p* for three-way interaction
Primary outcome: CASI total scores							
Baseline	75.3 ± 12.9	83.2 ± 7.7	Reference	78.0 ± 10.1	78.7 ± 11.3	Reference	-
2 weeks	70.6 ± 9.6	83.5 ± 9.9	−4.92 (−12.37, 2.53)	81.3 ± 9.9	80.4 ± 12.4	1.58 (−2.56, 5.72)	0.123
6 weeks	71.9 ± 12.6	84.1 ± 9.1	−4.32 (−11.77, 3.13)	82.7 ± 9.1	82.9 ± 13.4	0.50 (−3.64, 4.64)	0.251
14 weeks	74.6 ± 10.2	84.6 ± 8.5	−2.03 (−9.48, 5.42)	83.4 ± 9.8	82.6 ± 11.6	1.52 (−2.62, 5.66)	0.397
Secondary outcome*: WAIS-IV-vocabulary							
Baseline	20.8 ± 16.9	18.0 ± 9.4	Reference	15.7 ± 6.8	18.4 ± 10.8	Reference	-
2 weeks	18.0 ± 13.0	22.7 ± 8.9	−7.51 (−12.74, −2.29)	15.4 ± 7.4	17.5 ± 10.4	0.60 (−3.21, 4.42)	**0**.**028**
6 weeks	20.0 ± 14.4	22.3 ± 8.3	−5.09 (−10.31, 0.14)	16.1 ± 7.9	19.2 ± 11.8	−0.38 (−4.19, 3.44)	0.200
14 weeks	19.8 ± 15.3	24.1 ± 9.5	−7.14 (−12.37, −1.92)	16.4 ± 8.5	19.4 ± 12.5	−0.22 (−4.04, 3.59)	0.061

	MMSE ≥ 24 (*n* = 38)			MMSE < 24 (*n* = 12)			*P* value for three-way interaction
Variable	Sham (*n* = 20)	HD-tDCS (*n* = 18)	Group difference of the mean change from baseline (95% CI)	Sham (*n* = 5)	HD-tDCS (*n* = 7)	Group difference of the mean change from baseline (95% CI)	
Primary outcome: CASI total scores							
Baseline	80.9 ± 8.1	83.4 ± 8.7	Reference	64.5 ± 8.4	71.9 ± 9.8	Reference	-
2 weeks	83.1 ± 6.7	84.5 ± 9.1	1.02 (−2.80, 4.85)	62.9 ± 6.2	73.3 ± 13.3	−3.01 (−11.18, 5.16)	0.329
6 weeks	84.1 ± 6.0	87.4 ± 8.6	−0.88 (−4.70, 2.95)	65.0 ± 11.1	72.4 ± 12.7	0.01 (−8.16, 8.18)	0.830
14 weeks	84.8 ± 7.1	86.7 ± 7.6	0.48 (−3.35, 4.30)	67.8 ± 10.1	74.0 ± 11.7	1.24 (−6.93, 9.41)	0.853

*APOE4*: apolipoprotein E4 allele; CASI: Cognitive Abilities Screening Instrument; HD-tDCS: high definition transcranial direct current stimulation; MMSE: Mini-Mental State Examination; WASI: Wechsler Adult Intelligence Scale

Data are presented as frequency (percentage) or mean ± standard deviation.

Bold type indicates significant difference.

*Only significant differences were shown regarding secondary outcomes.

### Tolerability and integrity of blinding

The most common adverse events in the sham and active HD-tDCS groups included a tingling sensation (48.5% versus 56.9%), followed by skin redness (14.1% versus 18.8%) and burning sensation (7.7% versus 18%). No significant difference was observed in the percentage of adverse effects between the sham and active HD-tDCS groups (Supplemental Table 3). All these discomforts disappeared soon after the stimulation sessions. Two participants and one participant were dropped out in the sham and active HD-tDCS group, respectively. In both the sham and active HD-tDCS group, one participant was lost follow-up.

In the sham group, 48.0% correctly guessed that they had received sham HD-tDCS, whereas 30.8% of the participants in the active group correctly guessed that they had received active HD-tDCS. Chi-square tests evaluating blinding adequacy found no significant association between the participants’ guesses about their treatment (p = 0.16) (data not shown).

## Discussion

In the present triple-blind sham-controlled randomized controlled trial (RCT) applying HD-tDCS over the Lt DLPFC in a sufficiently powered sample of patients with MCI, we found (1) delayed improvement in global cognition in the 6-week and 14-week follow-up periods following daily 25 min session of HD-tDCS for 2 consecutive weeks. However, the effect of active stimulation was not superior to that of sham stimulation; (2) in subgroup analysis, MCI *APOE4*-positive patients who received HD-tDCS for 10 daily sessions have a potential improvement in language function than *APOE4*-negative patients receiving sham stimulation; (3) the HD-tDCS treatment was well tolerated.

The present study adopted a more focal stimulation therapy with HD-tDCS, which has not yet been sufficiently investigated. Previous studies have investigated the effects of HD-tDCS in patients with MCI, but the findings have been inconsistent. While some reported improvements in global cognition (measured using MoCA)^16,18^ and visuospatial subdomain cognitive function,^17^ others found nosignificant cognitive benefits.^19^ These inconsistent findings may be attributed to variations in study methodologies, such as the use of a single stimulation session,^17^ atypical montage configurations (cathodal over the Lt DLPFC surrounded by four anodal electrodes),^16^ small sample sizes ranging from as few as 20^17^ to 60 (with 20 participants per group),^16^ and the use of single-blind designs.^18^ Compared to previous research, we conducted a more comprehensive study with a triple-blind sham-controlled RCT trial with the application of a typical montage of anodal electrodes over F3 for 10 sessions in relatively large sample size (with 25 participants per group). However, we did not find any global cognitive benefits of HD-tDCS in patients with MCI. Therefore, the evidence of a positive effect of HD-tDCS on global cognitive functioning in these patients remains unclear.

Several explanations for the lack of cognitive benefit in HD-tDCS for patients with MCI could be addressed as follows. First, a meta-analysis of 17 studies using conventional tDCS in 616 patients with MCI and AD found that patients receiving more than 10 stimulation sessions had improved cognitive function compared to those receiving less than 10 sessions.^25^ The present study used only 10 daily stimulations; therefore, the duration of HD-tDCS might not have been sufficient. Second, patients with MCI are heterogeneous in their distinct neuropsychological profiles, neuroimaging characteristics (e.g., cortical thinning, white matter volume and integrity, and functional connectivity), and genetic factors. High between-patient variability may respond differently to HD-tDCS, resulting in variable synaptic plasticity and network reorganization. Notably, the highest level of evidence from meta-analyses has shown that conventional tDCS has cognitive benefits in patients with AD and MCI. However, the effect of tDCS on cognition disappeared in subgroup analyses that only included patients with MCI.^6,25^ One recent meta-analysis of eight RCTs of patients with MCI also confirmed that active tDCS treatment showed insignificant improvements in global cognition and several subdomains of cognition.^13^ Third, more than 50% of healthy participants had only a minor or no response in corticospinal excitability to tDCS over the motor cortex.^26^ The instability of the predicted action of tDCS may be more profound in patients with MCI, whose individual morphological differences have become more significant. Fourth, several cognitive regions are required to complete a cognitive test; thus, the stimulation of only one specific brain region may be less likely to modulate the brain network involved. Additionally, the effects of brain stimulation may be influenced by the state dependency of the targeted cortical regions,^27^ particularly in tDCS studies where the induced subthreshold polarization of cortical neurons is too weak to trigger action potential. The present study did not request participants to perform cognitive tasks while receiving HD-tDCS. The lack of “on-task” stimulation study design may explain the null findings to some extent. Taken together, the role of HD-tDCS in improving cognitive function in patients with MCI still awaits further replication and translation in clinical practice. Longer stimulation duration, identifying relatively homogenous MCI by incorporating biomarkers (neuropsychological characteristics, gene, and neuroimaging), application adequate “on task” HD-tDCS stimulation, and careful modeling studies to clarify interaction between polarity and neuronal orientation may be considered in the future studies.

In the subgroup analysis, we found that patients with MCI harboring the *APOE4* allele showed improved verbal memory after 10 daily sessions of HD-tDCS stimulation in the Lt DLPFC. We should point out that the inclusion of *APOE4* as a moderating variable was not part of the initial hypothesis or study design. We did not perform a predetermined sample size calculation specifically for the *APOE4* subgroup. Therefore, the findings should be considered as exploratory and hypothesis-generating. Even though, a population-based cohort study found that participants with the *APOE4* allele had a negative association with verbal episodic memory and showed rapid longitudinal decline.^28^ Tau imaging studies have shown that *APOE4* is associated with enhanced tau deposition in the medial temporal regions of the brain, which is vital for episodic memory.^29^ The present study found that the impact of HD-tDCS on verbal episodic memory in patients with MCI may differ depending on their *APOE4* status. A meta-analysis of 2025 older adults with at least one *APOE4* allele found that physical exercise had a greater effect on cognitive performance than in those without the *APOE4* allele.^30^ Some studies have suggested a potential mechanism of the *APOE4* allele mediating tDCS on cognitive function. First, previous studies have shown that conventional anodal tDCS appears to restore the local intrinsic change in the temporal pole, a part of the default mode network that is vulnerable to AD pathology, in patients with MCI.^31^ Second, the inhibitory network of GABAergic interneurons is responsible for the production of gamma oscillations linked to cognitive function.^32^ However, *APOE4* overexpression mice showed progressive degeneration of GABAergic interneurons, leading to slow gamma activity and cognitive impairment.^33^ Studies have shown that anodal tDCS increases visual gamma synchronization.^34^

A double-blind, randomized, crossover study of healthy participants showed that anodal tDCS over the Lt DLPFC significantly increased gamma-band oscillations.^35^ In summary, the mediating effect of *APOE4* on tDCS may be attributed alterations in the default mode network and gamma oscillation. However, the findings warrant further investigation to replicate.

The strength of this study lies in its novelty; to the best of our knowledge, this is the first triple-blind, sham-controlled study to assess the efficacy and safety of HD-tDCS in patients with MCI. We calculated the sample size to achieve at least a small effect size of HD-tDCS on global cognitive function, aiming to avoid potential type II errors. Furthermore, we subtyped patients with MCI based on their *APOE4* status and MMSE scores to improve the homogeneity and accuracy of the outcomes.

### Limitations

This study had some limitations. First, the study did not include MRI or electroencephalography to explore changes in brain networks or the neurophysiological mechanisms of efficacy. Second, blood and cerebrospinal fluid samples were not collected, making it impossible to investigate neurotransmitter changes. Third, we enrolled patients with clinically heterogeneous MCI. The use of biomarkers (e.g., brain imaging, blood, or cerebrospinal fluid testing) may enhance diagnostic specificity and treatment efficacy. Fourth, no long-term follow-up was conducted to assess the effectiveness of tDCS.

### Conclusions

The present study showed HD-tDCS did not improve global cognition in patients with MCI compared to sham groups. HD-tDCS is well-tolerated and good acceptability. Furthermore, we found a possible beneficial effect of HD-tDCS on language function in *APOE4* carriers with MCI, these findings should be interpreted as exploratory and hypothesis-generating. Rigorous, targeted studies are warranted to confirm the potential interaction between *APOE4* status and cognitive improvement following HD-tDCS.

### Supplemental Material

sj-docx-1-alz-10.1177_13872877251376547 - Supplemental material for Population-attributable fractions for modifiable risk factors across the continuum of cognitive decline: Hubei Memory and Aging Cohort Study Supplemental material, sj-docx-1-alz-10.1177_13872877251376547 for Population-attributable fractions for modifiable risk factors across the continuum of cognitive decline: Hubei Memory and Aging Cohort Study by Juan Zhou, Feifei Hu, Xiaochang Liu, Cheng Cai, Guirong Cheng, Xinyan Xie, Dan Liu, Deyang Zeng, Qianqian Nie, Jing Liu, JunyiWang, Shiyao Pan, Yuyang Cui, Dan Song, Shiyue Li, Jingjing Zhang, Wei Tan and Yan Zeng in Journal of Alzheimer's Disease
