# Supplementary material for: Effectiveness and safety of high-definition transcranial direct current stimulation in patients with mild cognitive impairment: A randomized, triple-blind, sham-controlled trial
Source: J Alzheimers Dis. 2025 Sep 22;108(1):298–311. doi: 10.1177/13872877251376547 (PMC13247333; doi:10.1177/13872877251376547)
Supplement: sj-docx-1-alz-10.1177_13872877251376547 - Supplemental material for Population-attributable fractions for modifiable risk factors across the continuum of cognitive decline: Hubei Memory and Aging Cohort Study [file sj-docx-1-alz-10.1177_13872877251376547.docx]

# **Supplemental Material**

# **Effectiveness and safety of high-definition transcranial direct current stimulation in patients with mild cognitive impairment: A randomized, triple-blind, sham-controlled trial**

# **Supplemental Table 1.** The results of linear mixed models between the sham stimulation and the active HD-tDCS groups

|  |  |  |  |  | Between time | |  |
| --- | --- | --- | --- | --- | --- | --- | --- |
| Variable | Sham  (*n* = 25) | HD-tDCS  (*n* = 25) | Group difference of the mean change from baseline (95% CI) | *p* for interaction | *p** of Sham | *p** of  HD-tDCS | *p* between group |
| WCST: total errors, % |  |  |  |  |  |  |  |
| Baseline | 83.2 ± 13.2 | 73.8 ± 22.9 | Reference | - | - | - | 0.128 |
| 2-week | 78.8 ± 15.4 | 71.9 ± 25.7 | -2.48 (-10.62, 5.66) | 0.548 | 0.136 | 0.519 | 0.261 |
| 6-week | 79.7 ± 20.3 | 71.5 ± 24.6 | -1.28 (-9.42, 6.86) | 0.756 | 0.229 | 0.443 | 0.188 |
| 14-week | 78.2 ± 19.5 | 74.2 ± 28.2 | -5.40 (-13.54, 2.74) | 0.192 | 0.088 | 0.891 | 0.513 |
| WCST: perseverative responses, % |  |  |  |  |  | **-** |  |
| Baseline | 44.0 ± 27.8 | 47.3 ± 33.1 | Reference | - | - | - | 0.725 |
| 2-week | 44.8 ± 31.1 | 48.6 ± 34.7 | -0.64 (-10.46, 9.18) | 0.898 | 0.838 | 0.699 | 0.673 |
| 6-week | 44.7 ± 33.0 | 48.1 ± 35.1 | -0.16 (-9.98, 9.66) | 0.974 | 0.856 | 0.820 | 0.712 |
| 14-week | 41.9 ± 32.6 | 45.1 ± 31.8 | 0.04 (-9.78, 9.86) | 0.994 | 0.547 | 0.540 | 0.728 |
| WCST: preservative errors, % |  |  |  |  |  | **-** |  |
| Baseline | 37.5 ± 19.6 | 39.3 ± 24.0 | Reference | - | - | - | 0.783 |
| 2-week | 37.8 ± 22.3 | 39.6 ± 25.1 | 0.00 (-7.24, 7.24) | 1.000 | 0.902 | 0.902 | 0.783 |
| 6-week | 37.6 ± 24.5 | 40.2 ± 25.3 | -0.80 (-8.04, 6.44) | 0.828 | 0.963 | 0.723 | 0.693 |
| 14-week | 35.6 ± 23.6 | 37.9 ± 23.6 | -0.48 (-7.72, 6.76) | 0.896 | 0.460 | 0.579 | 0.728 |
| WCST: non-perseverative errors, % |  |  |  |  |  | **-** |  |
| Baseline | 45.7 ± 20.0 | 34.5 ± 23.8 | Reference | - | - | **-** | 0.084 |
| 2-week | 41.0 ± 21.1 | 32.2 ± 22.1 | -2.44 (-10.12, 5.24) | 0.531 | 0.091 | 0.416 | 0.176 |
| 6-week | 42.1 ± 23.7 | 31.3 ± 21.6 | -0.44 (-8.12, 7.24) | 0.910 | 0.188 | 0.246 | 0.097 |
| 14-week | 42.6 ± 25.6 | 36.3 ± 24.6 | -4.88 (-12.56, 2.80) | 0.211 | 0.264 | 0.514 | 0.327 |

CASI: Cognitive Abilities Screening Instrument; WMS-III: Wechsler Memory Scale-Third Edition; WMS-IV: Wechsler Memory Scale-Fourth Edition; FAB: Frontal Assessment Battery.

Data were presented as mean ± standard deviation.

Bold type indicates significant difference.

*The contrast of simple main effect within the linear mixed model with two-way interactions.

# **Supplemental Table 2A.** Subgroup analysis: the results of linear mixed models in the sham stimulation and active HD-tDCS groups: *APOE4*+ and *APOE4*-

|  | *APOE4*+ (*n* = 12) | | | | *APOE4*- (*n* = 35) | | | |  | |
| --- | --- | --- | --- | --- | --- | --- | --- | --- | --- | --- |
| Variable | Sham  (*n* = 5) | HD-tDCS  (*n* = 7) | Group difference of the mean change from baseline (95% CI) | Sham  (*n* = 18) | | HD-tDCS  (*n* = 17) | Group difference of the mean change from baseline (95% CI) | *p* for three-way interaction | |  |
| WMS-III-Visual paired association-immediate | |  |  |  | |  |  |  | |  |
| Baseline | 0.0 ± 0.0 | 5.1 ± 7.4 | Reference | 2.2 ± 2.4 | | 4.8 ± 6.1 | Reference | - | |  |
| 2-week | 1.6 ± 2.1 | 7.4 ± 8.5 | -0.69 (-4.66, 3.29) | 4.6 ± 5.1 | | 6.9 ± 8.4 | 0.16 (-2.49, 2.81) | 0.744 | |  |
| 6-week | 3.2 ± 3.4 | 8.1 ± 9.6 | 0.20 (-3.77, 4.17) | 5.3 ± 4.8 | | 7.9 ± 8.4 | -0.12 (-2.77, 2.53) | 0.901 | |  |
| 14-week | 3.4 ± 5.0 | 8.4 ± 9.2 | 0.11 (-3.86, 4.09) | 6.3 ± 5.9 | | 9.4 ± 9.6 | -0.59 (-3.24, 2.06) | 0.785 | |  |
| WMS-III-visual reproduction-immediate | |  |  |  | |  |  |  | |  |
| Baseline | 10.8 ± 7.0 | 11.1 ± 3.0 | Reference | 12.3 ± 7.3 | | 11.8 ± 5.8 | Reference | - | |  |
| 2-week | 8.4 ± 4.4 | 15.1 ± 8.1 | -6.40 (-12.93, 0.13) | 15.7 ± 11.9 | | 14.8 ± 6.3 | 0.44 (-4.08, 4.97) | 0.120 | |  |
| 6-week | 10.0 ± 5.5 | 15.0 ± 4.2 | -4.66 (-11.19, 1.87) | 17.4 ± 9.8 | | 15.4 ± 8.0 | 1.52 (-3.01, 6.05) | 0.160 | |  |
| 14-week | 11.2 ± 4.9 | 13.9 ± 5.0 | -2.31 (-8.84, 4.22) | 18.3 ± 11.0 | | 16.4 ± 8.6 | 1.35 (-3.17, 5.88) | 0.403 | |  |
| WMS-III-Visual paired association-delay | |  |  |  | |  |  |  | |  |
| Baseline | 0.0 ± 0.0 | 1.0 ± 1.5 | Reference | 0.8 ± 1.1 | | 1.2 ± 2.0 | Reference | - | |  |
| 2-week | 0.0 ± 0.0 | 2.1 ± 2.5 | -1.14 (-2.88, 0.60) | 1.4 ± 1.8 | | 1.5 ± 2.4 | 0.37 (-0.60, 1.34) | 0.125 | |  |
| 6-week | 1.4 ± 2.2 | 2.1 ± 2.7 | 0.26 (-1.48, 2.00) | 1.6 ± 1.4 | | 2.5 ± 3.5 | -0.46 (-1.43, 0.51) | 0.465 | |  |
| 14-week | 1.4 ± 2.2 | 2.4 ± 2.8 | -0.03 (-1.77, 1.71) | 1.5 ± 1.9 | | 2.3 ± 2.3 | -0.40 (-1.37, 0.58) | 0.709 | |  |
| WMS-III-visual reproduction-delay |  |  |  |  | |  |  |  | |  |
| Baseline | 8.2 ± 7.2 | 8.4 ± 5.4 | Reference | 10.3 ± 6.6 | | 10.1 ± 7.7 | Reference | - | |  |
| 2-week | 8.2 ± 4.0 | 12.7 ± 8.8 | -4.29 (-12.42, 3.85) | 14.3 ± 11.4 | | 12.6 ± 9.2 | 1.47 (-3.72, 6.67) | 0.260 | |  |
| 6-week | 9.0 ± 4.8 | 11.7 ± 7.0 | -2.49 (-10.62, 5.65) | 14.1 ± 11.0 | | 15.4 ± 9.3 | -1.52 (-6.71, 3.68) | 0.849 | |  |
| 14-week | 6.0 ± 4.0 | 13.7 ± 4.9 | -7.49 (-15.62, 0.65) | 17.0 ± 13.1 | | 14.3 ± 8.7 | 2.49 (-2.70, 7.68) | 0.052 | |  |
| WAIS-IV-digit span |  |  |  |  | |  |  |  | |  |
| Baseline | 18.8 ± 5.4 | 21.3 ± 6.3 | Reference | 20.8 ± 5.1 | | 18.9 ± 5.6 | Reference | - | |  |
| 2-week | 20.2 ± 6.1 | 22.9 ± 7.0 | -0.17 (-4.89, 4.54) | 19.9 ± 5.2 | | 19.8 ± 6.6 | -1.83 (-3.81, 0.15) | 0.447 | |  |
| 6-week | 19.8 ± 7.4 | 22.3 ± 5.9 | 0.00 (-4.71, 4.71) | 20.4 ± 5.9 | | 20.6 ± 7.1 | -2.15 (-4.13, -0.17) | 0.324 | |  |
| 14-week | 22.0 ± 7.2 | 24.6 ± 5.5 | -0.09 (-4.80, 4.63) | 21.9 ± 5.8 | | 19.9 ± 7.0 | 0.11 (-1.87, 2.09) | 0.928 | |  |
| WAIS-IV-digit symbol coding |  |  |  |  | |  |  |  | |  |
| Baseline | 12.2 ± 7.2 | 18.6 ± 6.5 | Reference | 15.4 ± 8.3 | | 14.6 ± 8.7 | Reference | - | |  |
| 2-week | 13.8 ± 9.6 | 19.0 ± 10.9 | 1.17 (-6.51, 8.86) | 16.8 ± 10.0 | | 17.5 ± 8.5 | -1.49 (-4.50, 1.52) | 0.432 | |  |
| 6-week | 15.0 ± 10.9 | 18.9 ± 11.3 | 2.51 (-5.17, 10.20) | 16.9 ± 10.2 | | 18.8 ± 8.8 | -2.68 (-5.69, 0.33) | 0.127 | |  |
| 14-week | 15.2 ± 6.3 | 19.7 ± 7.1 | 1.86 (-5.83, 9.54) | 17.7 ± 9.3 | | 19.6 ± 10.1 | -2.66 (-5.67, 0.35) | 0.184 | |  |
| WCST: total trials |  |  |  |  | |  |  |  | |  |
| Baseline | 42.0 ± 18.4 | 44.7 ± 12.3 | Reference | 45.7 ± 12.6 | | 52.0 ± 16.8 | Reference | - | |  |
| 2-week | 36.4 ± 7.2 | 46.0 ± 14.5 | -6.89 (-25.32, 11.54) | 53.3 ± 15.8 | | 52.2 ± 17.8 | 7.43 (-1.15, 16.02) | 0.118 | |  |
| 6-week | 43.6 ± 26.5 | 47.7 ± 18.4 | -1.40 (-19.83, 17.03) | 49.3 ± 18.1 | | 53.2 ± 17.5 | 2.49 (-6.09, 11.07) | 0.669 | |  |
| 14-week | 40.8 ± 22.6 | 40.7 ± 8.8 | 2.80 (-15.63, 21.23) | 52.9 ± 19.4 | | 48.1 ± 16.6 | 11.22 (2.64, 19.80) | 0.356 | |  |
| WCST: total errors, % |  |  |  |  | |  |  |  | |  |
| Baseline | 86.0 ± 18.4 | 83.1 ± 12.3 | Reference | 82.3 ± 12.6 | | 73.0 ± 22.7 | Reference | - | |  |
| 2-week | 91.6 ± 7.2 | 82.0 ± 14.5 | 6.74 (-12.80, 26.28) | 74.7 ± 15.8 | | 70.7 ± 26.6 | -5.32 (-15.13, 4.50) | 0.238 | |  |
| 6-week | 82.0 ± 31.8 | 80.3 ± 18.4 | -1.14 (-20.68, 18.40) | 78.6 ± 18.4 | | 70.9 ± 24.3 | -1.66 (-11.48, 8.16) | 0.960 | |  |
| 14-week | 87.2 ± 22.6 | 87.3 ± 8.8 | -2.94 (-22.48, 16.60) | 75.1 ± 19.4 | | 71.9 ± 30.1 | -6.16 (-15.98, 3.66) | 0.752 | |  |
| WCST: perseverative responses, % |  |  |  |  | |  |  |  | |  |
| Baseline | 34.6 ± 32.3 | 69.4 ± 40.1 | Reference | 46.1 ± 27.7 | | 40.2 ± 26.4 | Reference | - | |  |
| 2-week | 42.0 ± 38.4 | 71.4 ± 43.7 | 5.40 (-20.80, 31.60) | 45.0 ± 31.2 | | 41.4 ± 26.9 | -2.23 (-13.67, 9.21) | 0.538 | |  |
| 6-week | 38.8 ± 42.5 | 71.9 ± 41.1 | 1.77 (-24.43, 27.97) | 45.8 ± 32.6 | | 40.4 ± 28.7 | -0.45 (-11.90, 10.99) | 0.858 | |  |
| 14-week | 43.4 ± 45.9 | 72.6 ± 28.4 | 5.66 (-20.55, 31.86) | 40.7 ± 31.0 | | 35.7 ± 26.9 | -0.92 (-12.36, 10.52) | 0.596 | |  |
| WCST: preservative errors, % |  |  |  |  | |  |  |  | |  |
| Baseline | 30.0 ± 22.1 | 55.9 ± 27.7 | Reference | 39.1 ± 19.4 | | 34.2 ± 19.4 | Reference | - | |  |
| 2-week | 35.4 ± 27.6 | 56.1 ± 30.5 | 5.11 (-13.24, 23.47) | 38.0 ± 22.3 | | 34.5 ± 19.9 | -1.41 (-10.04, 7.22) | 0.476 | |  |
| 6-week | 32.4 ± 31.1 | 57.6 ± 28.1 | 0.69 (-17.67, 19.04) | 38.6 ± 24.1 | | 34.8 ± 21.2 | -1.15 (-9.78, 7.48) | 0.841 | |  |
| 14-week | 35.6 ± 32.2 | 59.0 ± 19.0 | 2.46 (-15.90, 20.82) | 34.8 ± 22.6 | | 30.8 ± 20.2 | -0.81 (-9.44, 7.82) | 0.721 | |  |
| WCST: non-perseverative errors, % |  |  |  |  | |  |  |  | |  |
| Baseline | 56.0 ± 25.5 | 27.4 ± 24.2 | Reference | 43.3 ± 19.0 | | 38.8 ± 23.4 | Reference | - | |  |
| 2-week | 56.2 ± 29.3 | 25.9 ± 19.5 | 1.77 (-17.84, 21.38) | 36.7 ± 18.1 | | 36.2 ± 22.9 | -3.91 (-13.10, 5.28) | 0.560 | |  |
| 6-week | 49.6 ± 35.0 | 22.7 ± 22.6 | -1.69 (-21.29, 17.92) | 40.0 ± 21.7 | | 36.1 ± 20.7 | -0.51 (-9.70, 8.67) | 0.904 | |  |
| 14-week | 51.6 ± 36.8 | 28.3 ± 21.5 | -5.26 (-24.86, 14.35) | 40.2 ± 23.8 | | 41.1 ± 25.2 | -5.35 (-14.54, 3.84) | 0.992 | |  |
| Frontal assessment battery |  |  |  |  | |  |  |  | |  |
| Baseline | 10.6 ± 3.7 | 12.3 ± 2.4 | Reference | 11.4 ± 3.3 | | 11.9 ± 2.9 | Reference | - | |  |
| 2-week | 11.6 ± 3.8 | 12.7 ± 1.8 | 0.57 (-1.74, 2.88) | 11.3 ± 2.8 | | 12.1 ± 3.1 | -0.23 (-1.70, 1.24) | 0.581 | |  |
| 6-week | 11.6 ± 3.8 | 13.3 ± 2.1 | 0.00 (-2.31, 2.31) | 12.8 ± 2.7 | | 12.6 ± 3.2 | 0.69 (-0.79, 2.16) | 0.636 | |  |
| 14-week | 11.8 ± 2.9 | 14.0 ± 2.6 | -0.51 (-2.83, 1.80) | 12.6 ± 2.9 | | 12.9 ± 3.1 | 0.11 (-1.36, 1.58) | 0.666 | |  |
| BDI-II |  |  |  |  | |  |  |  | |  |
| Baseline | 6.2 ± 7.6 | 5.4 ± 4.7 | Reference | 5.7 ± 9.6 | | 3.1 ± 4.3 | Reference | - | |  |
| 2-week | 4.8 ± 8.7 | 3.0 ± 5.1 | 1.03 (-3.75, 5.80) | 3.7 ± 5.6 | | 3.0 ± 4.0 | -2.00 (-4.71, 0.71) | 0.269 | |  |
| 6-week | 3.2 ± 6.6 | 2.6 ± 4.8 | -0.14 (-4.92, 4.63) | 3.9 ± 5.2 | | 3.7 ± 4.6 | -2.48 (-5.19, 0.23) | 0.392 | |  |
| 14-week | 4.0 ± 7.3 | 5.0 ± 6.1 | -1.77 (-6.55, 3.00) | 4.2 ± 5.8 | | 2.5 ± 3.6 | -0.97 (-3.68, 1.74) | 0.769 | |  |
| BAI |  |  |  |  | |  |  |  | |  |
| Baseline | 7.2 ± 11.2 | 2.1 ± 2.0 | Reference | 5.1 ± 7.2 | | 3.0 ± 4.5 | Reference | - | |  |
| 2-week | 4.2 ± 6.6 | 1.4 ± 2.1 | -2.29 (-8.14, 3.57) | 3.3 ± 5.5 | | 1.6 ± 3.6 | -0.37 (-2.40, 1.67) | 0.426 | |  |
| 6-week | 1.8 ± 2.9 | 2.4 ± 4.4 | -5.69 (-11.54, 0.17) | 2.6 ± 3.6 | | 1.5 ± 2.8 | -1.03 (-3.06, 1.00) | 0.055 | |  |
| 14-week | 4.2 ± 5.7 | 2.9 ± 4.3 | -3.71 (-9.57, 2.14) | 3.0 ± 3.9 | | 1.4 ± 2.5 | -0.41 (-2.44, 1.62) | 0.171 | |  |
| SCD-Q score |  |  |  |  | |  |  |  | |  |
| Baseline | 6.8 ± 6.2 | 8.7 ± 3.6 | Reference | 8.4 ± 3.4 | | 7.5 ± 3.8 | Reference | - | |  |
| 2-week | 8.0 ± 5.5 | 7.6 ± 3.6 | 2.34 (-0.99, 5.68) | 8.3 ± 4.0 | | 6.7 ± 3.3 | 0.71 (-1.32, 2.74) | 0.419 | |  |
| 6-week | 7.4 ± 5.9 | 7.7 ± 4.0 | 1.60 (-1.73, 4.93) | 7.8 ± 3.8 | | 7.3 ± 3.8 | -0.32 (-2.35, 1.71) | 0.341 | |  |
| 14-week | 5.8 ± 5.0 | 7.6 ± 3.9 | 0.14 (-3.19, 3.48) | 7.9 ± 3.9 | | 8.6 ± 3.4 | -1.56 (-3.59, 0.47) | 0.399 | |  |

# **Supplemental Table 2B.** Subgroup analysis: the results of linear mixed models in the sham stimulation and active HD-tDCS groups: MMSE≥24 and MMSE<24

|  | MMSE≥24 (*n* = 38) | | | MMSE<24 (*n* = 12) | | |  |
| --- | --- | --- | --- | --- | --- | --- | --- |
| Variable | Sham  (*n* = 20) | HD-tDCS  (*n* = 18) | Group difference of the mean change from baseline (95% CI) | Sham  (*n* = 5) | HD-tDCS  (*n* = 7) | Group difference of the mean change from baseline (95% CI) | *p* for three-way interaction |
| WMS-III-visual paired association-immediate | |  |  |  |  |  |  |
| Baseline | 2.7 ± 3.5 | 6.9 ± 6.7 | Reference | 0.0 ± 0.0 | 0.4 ± 0.5 | Reference | - |
| 2-week | 5.2 ± 5.2 | 9.7 ± 8.4 | -0.27 (-2.81, 2.27) | 0.2 ± 0.4 | 1.0 ± 1.0 | -0.37 (-2.26, 1.52) | 0.967 |
| 6-week | 6.1 ± 4.8 | 10.6 ± 8.5 | -0.27 (-2.81, 2.28) | 0.6 ± 0.9 | 1.7 ± 3.0 | -0.69 (-2.58, 1.20) | 0.861 |
| 14-week | 7.2 ± 5.7 | 12.3 ± 8.9 | -0.83 (-3.38, 1.71) | 0.0 ± 0.0 | 1.3 ± 1.7 | -0.86 (-2.75, 1.03) | 0.992 |
| WMS-III-visual reproduction-immediate | |  |  |  |  |  |  |
| Baseline | 12.2 ± 7.6 | 12.0 ± 5.5 | Reference | 9.8 ± 4.7 | 9.1 ± 4.6 | Reference | - |
| 2-week | 15.3 ± 11.4 | 15.2 ± 7.2 | -0.07 (-4.22, 4.09) | 7.4 ± 5.6 | 12.3 ± 6.8 | -5.54 (-11.94, 0.85) | 0.191 |
| 6-week | 16.5 ± 10.0 | 16.5 ± 7.5 | -0.20 (-4.36, 3.96) | 10.4 ± 5.4 | 10.3 ± 4.9 | -0.54 (-6.94, 5.85) | 0.935 |
| 14-week | 17.4 ± 10.9 | 17.5 ± 7.8 | -0.35 (-4.51, 3.81) | 11.2 ± 5.6 | 9.0 ± 5.1 | 1.54 (-4.85, 7.94) | 0.651 |
| WMS-III-visual paired association-delay | |  |  |  |  |  |  |
| Baseline | 0.9 ± 1.3 | 1.6 ± 2.0 | Reference | 0.0 ± 0.0 | 0.1 ± 0.4 | Reference | - |
| 2-week | 1.5 ± 1.8 | 2.2 ± 2.6 | -0.07 (-1.08, 0.94) | 0.0 ± 0.0 | 0.3 ± 0.5 | -0.14 (-0.71, 0.42) | 0.936 |
| 6-week | 2.0 ± 1.6 | 3.1 ± 3.4 | -0.46 (-1.47, 0.56) | 0.0 ± 0.0 | 0.4 ± 1.1 | -0.29 (-0.85, 0.28) | 0.857 |
| 14-week | 1.9 ± 2.0 | 3.1 ± 2.3 | -0.50 (-1.51, 0.51) | 0.0 ± 0.0 | 0.4 ± 0.8 | -0.29 (-0.85, 0.28) | 0.820 |
| WMS-III-visual reproduction-delay |  |  |  |  |  |  |  |
| Baseline | 10.8 ± 7.1 | 10.7 ± 7.4 | Reference | 6.0 ± 4.6 | 5.9 ± 4.7 | Reference | - |
| 2-week | 14.1 ± 11.1 | 14.3 ± 9.0 | -0.37 (-5.50, 4.77) | 7.0 ± 4.0 | 6.7 ± 6.6 | 0.14 (-5.91, 6.19) | 0.919 |
| 6-week | 14.2 ± 10.7 | 16.3 ± 8.8 | -2.27 (-7.40, 2.87) | 6.8 ± 4.3 | 7.4 ± 5.7 | -0.77 (-6.82, 5.28) | 0.764 |
| 14-week | 15.9 ± 13.2 | 15.3 ± 8.0 | 0.43 (-4.70, 5.57) | 7.4 ± 5.0 | 9.3 ± 6.2 | -2.03 (-8.08, 4.02) | 0.622 |
| WAIS-IV-digit span |  |  |  |  |  |  |  |
| Baseline | 21.2 ± 4.5 | 20.2 ± 5.7 | Reference | 15.8 ± 5.1 | 18.7 ± 6.2 | Reference | - |
| 2-week | 20.7 ± 4.8 | 21.9 ± 6.2 | -2.12 (-4.06, -0.18) | 15.6 ± 4.8 | 18.1 ± 7.3 | 0.37 (-3.42, 4.16) | 0.225 |
| 6-week | 21.4 ± 5.3 | 22.9 ± 6.1 | -2.42 (-4.36, -0.48) | 14.2 ± 5.2 | 17.0 ± 6.2 | 0.11 (-3.68, 3.91) | 0.217 |
| 14-week | 23.0 ± 5.4 | 22.2 ± 6.0 | -0.20 (-2.14, 1.74) | 15.8 ± 4.5 | 19.3 ± 8.5 | -0.57 (-4.36, 3.22) | 0.856 |
| WAIS-IV-vocabulary |  |  |  |  |  |  |  |
| Baseline | 19.7 ± 9.8 | 19.3 ± 10.5 | Reference | 7.6 ± 1.8 | 14.6 ± 8.8 | Reference | - |
| 2-week | 18.5 ± 9.2 | 19.4 ± 10.5 | -1.37 (-5.09, 2.36) | 8.6 ± 1.3 | 16.7 ± 9.0 | -1.14 (-4.92, 2.63) | 0.950 |
| 6-week | 19.7 ± 9.7 | 21.4 ± 11.0 | -2.17 (-5.89, 1.56) | 8.4 ± 2.3 | 15.3 ± 9.1 | 0.09 (-3.69, 3.86) | 0.529 |
| 14-week | 19.8 ± 10.6 | 21.8 ± 12.3 | -2.46 (-6.18, 1.27) | 9.0 ± 1.4 | 16.6 ± 9.5 | -0.60 (-4.37, 3.17) | 0.604 |
| WAIS-IV-digit symbol coding |  |  |  |  |  |  |  |
| Baseline | 16.6 ± 7.2 | 16.4 ± 8.5 | Reference | 4.4 ± 2.9 | 14.4 ± 6.9 | Reference | - |
| 2-week | 18.3 ± 8.8 | 19.8 ± 8.2 | -1.58 (-4.56, 1.39) | 4.0 ± 3.8 | 13.3 ± 9.2 | 0.74 (-5.59, 7.08) | 0.468 |
| 6-week | 19.0 ± 8.8 | 20.4 ± 9.7 | -1.54 (-4.52, 1.43) | 3.0 ± 3.7 | 14.7 ± 6.6 | -1.69 (-8.02, 4.65) | 0.965 |
| 14-week | 19.1 ± 7.7 | 21.6 ± 9.6 | -2.56 (-5.54, 0.41) | 5.4 ± 3.9 | 14.4 ± 4.7 | 1.00 (-5.33, 7.33) | 0.267 |
| WCST: total trials |  |  |  |  |  |  |  |
| Baseline | 47.8 ± 13.0 | 52.3 ± 17.2 | Reference | 32.8 ± 3.3 | 46.6 ± 12.2 | Reference | - |
| 2-week | 52.2 ± 15.5 | 55.7 ± 17.2 | 0.96 (-7.84, 9.77) | 37.2 ± 8.0 | 39.6 ± 10.0 | 11.40 (-0.64, 23.44) | 0.232 |
| 6-week | 52.0 ± 18.8 | 54.0 ± 17.2 | 2.53 (-6.27, 11.34) | 30.8 ± 4.7 | 48.1 ± 19.5 | -3.57 (-15.61, 8.47) | 0.483 |
| 14-week | 54.2 ± 19.0 | 49.9 ± 16.8 | 8.79 (-0.01, 17.60) | 32.4 ± 10.4 | 39.3 ± 7.7 | 6.89 (-5.15, 18.93) | 0.826 |
| WCST: total errors, % |  |  |  |  |  |  |  |
| Baseline | 80.2 ± 13.0 | 70.8 ± 25.6 | Reference | 95.2 ± 3.3 | 81.3 ± 12.2 | Reference | - |
| 2-week | 75.9 ± 15.5 | 65.4 ± 27.2 | 1.04 (-8.94, 11.02) | 90.8 ± 8.0 | 88.4 ± 10.0 | -11.54 (-23.51, 0.42) | 0.197 |
| 6-week | 75.3 ± 20.4 | 68.3 ± 26.1 | -2.34 (-12.33, 7.64) | 97.2 ± 4.7 | 79.9 ± 19.5 | 3.43 (-8.53, 15.39) | 0.553 |
| 14-week | 73.9 ± 19.0 | 68.5 ± 31.4 | -4.02 (-14.00, 5.97) | 95.6 ± 10.4 | 88.7 ± 7.7 | -7.03 (-18.99, 4.93) | 0.757 |
| WCST: perseverative responses, % |  |  |  |  |  |  |  |
| Baseline | 46.1 ± 29.2 | 41.9 ± 30.5 | Reference | 35.8 ± 21.8 | 61.1 ± 37.8 | Reference | - |
| 2-week | 46.6 ± 33.6 | 42.5 ± 30.4 | -0.16 (-10.88, 10.56) | 37.6 ± 19.4 | 64.4 ± 42.1 | -1.49 (-27.20, 24.23) | 0.912 |
| 6-week | 47.5 ± 35.3 | 40.8 ± 30.9 | 2.46 (-8.26, 13.18) | 33.6 ± 20.6 | 66.9 ± 40.5 | -7.91 (-33.63, 17.80) | 0.386 |
| 14-week | 44.8 ± 34.8 | 37.3 ± 28.8 | 3.31 (-7.41, 14.03) | 30.4 ± 21.1 | 65.3 ± 32.1 | -9.54 (-35.26, 16.17) | 0.283 |
| WCST: preservative errors, % |  |  |  |  |  |  |  |
| Baseline | 39.0 ± 20.4 | 35.1 ± 22.4 | Reference | 31.6 ± 16.4 | 50.1 ± 26.2 | Reference | - |
| 2-week | 38.8 ± 24.0 | 34.8 ± 22.6 | 0.13 (-7.93, 8.20) | 34.0 ± 15.2 | 52.1 ± 28.6 | 0.40 (-17.63, 18.43) | 0.976 |
| 6-week | 39.4 ± 26.2 | 34.6 ± 22.5 | 0.90 (-7.17, 8.97) | 30.6 ± 16.1 | 54.7 ± 28.1 | -5.57 (-23.60, 12.46) | 0.463 |
| 14-week | 37.5 ± 25.1 | 31.7 ± 21.5 | 1.99 (-6.07, 10.06) | 27.8 ± 16.5 | 53.9 ± 22.5 | -7.51 (-25.55, 10.52) | 0.281 |
| WCST: non-perseverative errors, % |  |  |  |  |  |  |  |
| Baseline | 41.3 ± 17.8 | 35.7 ± 25.1 | Reference | 63.6 ± 19.7 | 31.3 ± 21.4 | Reference | - |
| 2-week | 37.1 ± 19.7 | 30.7 ± 21.7 | 0.91 (-7.54, 9.35) | 56.8 ± 20.8 | 36.3 ± 24.2 | -11.80 (-29.72, 6.12) | 0.163 |
| 6-week | 36.0 ± 20.8 | 33.7 ± 20.1 | -3.24 (-11.69, 5.20) | 66.6 ± 19.4 | 25.1 ± 25.8 | 9.14 (-8.78, 27.07) | 0.174 |
| 14-week | 36.4 ± 22.9 | 36.8 ± 25.3 | -6.01 (-14.45, 2.43) | 67.8 ± 21.3 | 34.9 ± 24.4 | 0.63 (-17.29, 18.55) | 0.465 |
| Frontal assessment battery |  |  |  |  |  |  |  |
| Baseline | 12.2 ± 2.7 | 12.8 ± 2.0 | Reference | 7.6 ± 3.0 | 10.3 ± 3.4 | Reference | - |
| 2-week | 12.4 ± 1.6 | 12.8 ± 2.3 | 0.20 (-1.08, 1.48) | 7.4 ± 3.6 | 11.0 ± 3.3 | -0.91 (-3.74, 1.91) | 0.423 |
| 6-week | 13.4 ± 2.1 | 13.1 ± 2.3 | 0.87 (-0.41, 2.15) | 8.8 ± 3.0 | 12.1 ± 4.1 | -0.66 (-3.48, 2.17) | 0.272 |
| 14-week | 13.3 ± 2.2 | 13.9 ± 2.7 | -0.01 (-1.28, 1.27) | 8.6 ± 1.7 | 11.7 ± 3.0 | -0.43 (-3.25, 2.39) | 0.761 |
| BDI-II score |  |  |  |  |  |  |  |
| Baseline | 6.9 ± 10.1 | 3.9 ± 4.7 | Reference | 4.0 ± 5.7 | 3.1 ± 3.8 | Reference | - |
| 2-week | 4.6 ± 6.9 | 3.8 ± 4.6 | -2.19 (-4.86, 0.48) | 4.4 ± 8.8 | 0.9 ± 0.9 | 2.69 (-0.93, 6.30) | 0.066 |
| 6-week | 4.6 ± 6.7 | 4.4 ± 4.9 | -2.74 (-5.42, -0.07)* | 3.6 ± 6.5 | 0.7 ± 1.3 | 2.03 (-1.59, 5.64) | 0.072 |
| 14-week | 4.9 ± 7.0 | 3.8 ± 4.5 | -1.78 (-4.45, 0.89) | 4.2 ± 7.2 | 1.9 ± 4.1 | 1.49 (-2.13, 5.10) | 0.216 |
| BAI score |  |  |  |  |  |  |  |
| Baseline | 4.9 ± 6.8 | 2.6 ± 4.1 | Reference | 7.4 ± 11.1 | 2.9 ± 3.1 | Reference | - |
| 2-week | 3.1 ± 5.1 | 1.6 ± 3.6 | -0.75 (-2.83, 1.33) | 5.0 ± 6.7 | 1.3 ± 1.1 | -0.83 (-5.78, 4.12) | 0.973 |
| 6-week | 2.3 ± 3.4 | 2.0 ± 3.7 | -1.99 (-4.08, 0.09) | 3.2 ± 3.5 | 1.0 ± 1.0 | -2.34 (-7.30, 2.61) | 0.880 |
| 14-week | 3.5 ± 4.5 | 2.0 ± 3.5 | -0.79 (-2.88, 1.29) | 2.4 ± 2.7 | 1.0 ± 1.5 | -3.14 (-8.10, 1.81) | 0.311 |
| SCD-Q score |  |  |  |  |  |  |  |
| Baseline | 8.2 ± 4.1 | 8.6 ± 4.1 | Reference | 8.8 ± 4.1 | 7.0 ± 2.6 | Reference | - |
| 2-week | 8.2 ± 4.3 | 7.4 ± 3.9 | 1.12 (-0.75, 2.98) | 9.8 ± 4.0 | 6.9 ± 2.8 | 1.14 (-2.31, 4.60) | 0.989 |
| 6-week | 7.5 ± 4.4 | 7.8 ± 4.1 | -0.03 (-1.89, 1.83) | 10.4 ± 2.3 | 7.3 ± 3.9 | 1.31 (-2.14, 4.77) | 0.489 |
| 14-week | 7.6 ± 4.4 | 8.9 ± 3.5 | -0.93 (-2.80, 0.93) | 8.4 ± 3.7 | 7.6 ± 4.1 | -0.97 (-4.43, 2.49) | 0.984 |

HD-tDCS: high-definition transcranial direct current stimulation; *APOE4*: apolipoprotein E4 allele; CASI: cognitive abilities screening instrument; WMS-III: Wechsler memory scale-third Edition; WMS-IV: Wechsler memory scale-fourth edition; WCST: Wisconsin card sorting test; BDI: Beck depression inventory; BAI: Beck anxiety inventory; SCD-Q: subjective cognitive decline questionnaire.

Data were presented as mean ± standard deviation

**Supplemental Table 3.** Side effect reported during stimulation

|  | **Sham** | **HD-tDCS** | **p** |
| --- | --- | --- | --- |
| Tingling | 114/234=48.7% | 136/239=56.9% | 0.457 |
| Skin redness | 33/234=14.1% | 45/239=18.8% | 0.580 |
| Burn | 18/234=7.7% | 43/239=18% | 0.176 |
| Scalp pain | 15/234=6.4% | 11/239=4.6% | 0.738 |
| Itching | 11/234=4.7% | 26/239=10.9% | 0.285 |
| Burning sensation | 8/234=3.4% | 27/239=11.3% | 0.218 |
| Sleeping | 4/234=1.7% | 14/239=5.9% | 0.243 |
| Trouble concentrating | 4/234=1.7% | 4/239=1.7% | 1.000 |
| Acute mood change | 2/234=0.9% | 0/239=0% | 0.161 |
| Total | 234 | 239 |  |

HD-tDCS: high-definition transcranial direct current stimulation
